# Death of Prof. Agassiz

**Published:** 1874-01

**Authors:** 


*Death of Prof. Agassiz.*

There is not one of our readers perhaps, who is not already aware of the death of this eminent Scientist, and who does not feel that in his death science has lost one of its most illustrious devotees, and the world one of its best friends and greatest benefactors. What reading American has not heard of the great naturalist who lived in Boston, but who belonged to the world? In all cultivated families his name has been as a house-hold word, by the side of Scott, Dickens, and other authors in the world of polite and romantic literature.

No mere man of science in the new world or the old, ever attained such a wide celebrity among the masses of the people.

Born at Motiers, Switzerland, May 28th, 1807, he was early designed for the profession of medicine and pursued his studies to that end; but becoming, before their completion, interested in Natural History studies, particularly Ichthyology, he abandoned medicine as a profession, and bent all his energies to the pursuit of his favorite study and the collateral branches of Natural Science. It is not our purpose here to treat of his numerous discoveries, and additions to the store-house of scientific knowledge. They are now a part of the scientific history of the world, and are to be found in the books and periodicals devoted to science. He was a man of a large and well trained mind, with powers of observation naturally great, and sharpened by careful practice, an intense love for his study, and an unbounded enthusiasm in its pursuit. He possessed the essentials of an original investigator, which his assiduity and industry developed to the highest degree. As a generalizer of facts, however, his position is not so high as some others. That faculty was not his in an eminent degree, and hence, he failed to agree with the deductions of some of the first philosophical scientists of the day. His opposition to the evolution theory or Darwinism as it is commonly called, is well known. Indeed, a part of the work he had laid out for the coming year, was a series of articles against this rapidly spreading philosophy. In his death the anti-evolutionists have lost the only prominent scientist ,who has withstood the influence of the rapidly accumulating proofs in favor of this asfcinating theory. S. M. B.

